# Gut microbial metabolites in colorectal cancer: dual roles in tumorigenesis, immune crosstalk, and therapeutic innovation

**DOI:** 10.3389/fcimb.2026.1693161

**Published:** 2026-05-28

**Authors:** Yutao Jin, He Zhu, Jiawei Fan, Hong Xu

**Affiliations:** Department of Gastroenterology, The First Hospital of Jilin University, Changchun, China

**Keywords:** amino acids and amino acid-related metabolites, bile acids, colorectal cancer, metabolites, short-chain fatty acid

## Abstract

A substantial body of evidence has elucidated the critical role of gut microbiota in the development and progression of colorectal cancer (CRC). Gut dysbiosis, defined as the disruption of microbiome homeostasis, has been implicated in the pathogenesis of various diseases, including CRC, Parkinson’s disease, and autoimmune liver disorders. In recent years, research has increasingly focused on microbial metabolites, with numerous studies confirming their association with CRC. This review systematically elucidates the dual roles of microbial metabolites in the initiation and progression of CRC: they can suppress tumors by strengthening the gut barrier, reducing inflammation, blocking abnormal cell growth, and triggering apoptosis; yet under dysbiotic conditions-like chronic inflammation or epithelial injury-they may promote cancer by releasing inflammatory cytokines, damaging DNA, and driving uncontrolled proliferation. We summarize key findings on these metabolites’ functions in CRC, highlight emerging metabolite-targeted therapies, and identify major hurdles to clinical translation: metabolite instability, individual variation in host-microbe interactions, and absent biomarkers for patient selection. Because the gut microbiota-metabolite axis is central to CRC biology, targeting it rationally offers a promising path to more precise and effective treatments. Ultimately, gut metabolites are not just disease indicators-they are actionable therapeutic targets.

## 1 Introduction

CRC is one of the most prevalent malignancies globally. According to the 2022 data from the World Health Organization’s International Agency for Research on Cancer, CRC is the third most commonly diagnosed cancer worldwide, accounting for over 1.9 million new cases and approximately 900,000 deaths annually (Bray et al., 2024), ranking third in morbidity and second in mortality, following lung and breast cancers. The pathogenesis and progression of CRC involve multiple contributing factors, with gut microbiota playing a significant role.

The human intestine constitutes a highly complex ecosystem hosting millions of microorganisms, including bacteria, archaea, fungi, protists, and viruses (Adak and Khan, 2019). In the large intestine, anaerobic bacteria outnumber aerobic bacteria by a factor of 100 to 1000, with predominant genera such as *Bacteroides*, *Bifidobacterium*, *Streptococcus*, *Enterobacteriaceae*, *Enterococcus*, *Clostridium*, *Lactobacillus*, and *Akkermansia*, along with low-abundance pathogens like *Campylobacter jejuni*, *Vibrio cholerae*, *Salmonella*, *Escherichia coli*, and *Bacteroides fragilis* (Gou et al., 2023). Gut dysbiosis involving these microbial groups has been linked to CRC in the past decade (Cui et al., 2024). In healthy individuals, the gut microbiota is primarily composed of *Bacteroidetes* and *Firmicutes* (about 90%), with the rest including *Actinobacteria*, *Proteobacteria*, *Clostridium*, and *Verrucomicrobia* (Okumura et al., 2021). Patients with CRC exhibit significant alterations in fecal bacterial abundance, impacting at least 18 species (Li et al., 2022). Key microbial metabolites, including short-chain fatty acids (SCFAs), secondary bile acids, lipopolysaccharides (LPS), and tryptophan derivatives are closely associated with CRC development. SCFAs, such as butyrate and propionate reduce tumor growth through key mechanisms including HDAC inhibition, GPCR activation, and enhanced CD8^+^ T cell immunity. Butyrate has opposing effects depending on its concentration, with low levels potentially promoting cancer, while high levels suppress tumors, illustrating the complex role of microbial metabolites. Secondary bile acids (e.g., deoxycholic acid) and LPS promote CRC by activating Wnt/β-catenin and NF-κB pathways and causing DNA damage. In contrast, ursodeoxycholic acid can inhibit tumor growth via the GPR5-cAMP-PKA pathway. LPS may also improve clinical outcomes by modulating TOPORS expression. Tryptophan metabolites have diverse roles in CRC as well, for instance, 5-methoxytryptamine blocks CRC cell proliferation, whereas kynurenine and indole-3-acrylic acid (IDA) promote disease progression. Overall, gut microbial metabolites influence both tumor development and immunity in a context-dependent manner.

**Figure 1:**
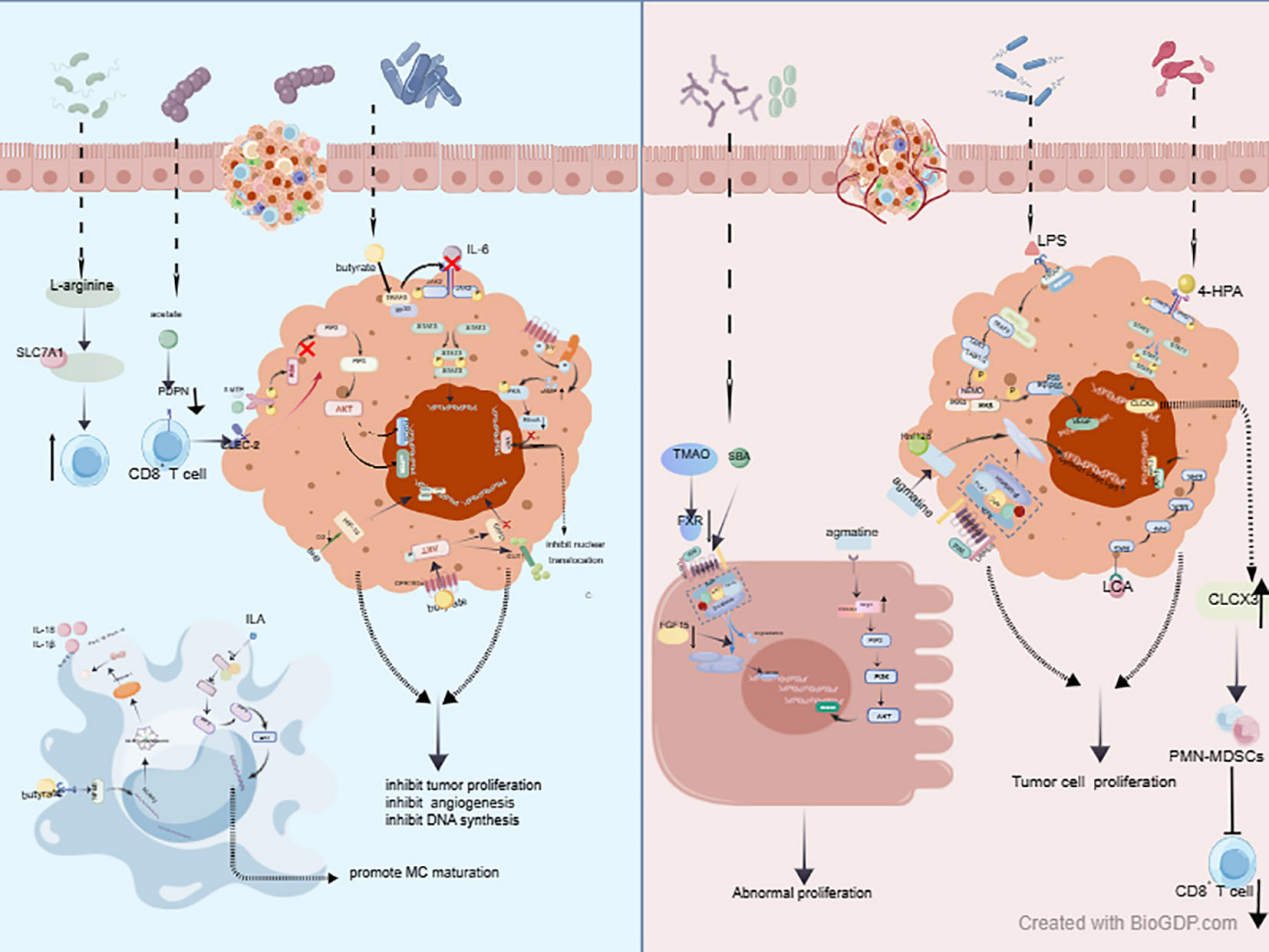
Bidirectional Regulatory Network of Tumor Promoting and Tumor Suppressive Microbial Metabolites in Colorectal Cancer Progression.

This review outlines how gut microbial metabolites influence CRC development, immunity, and related diseases **Figure 1**. It summarizes recent therapeutic strategies targeting these metabolites. By integrating current evidence, the review provides a practical framework for improving CRC diagnosis and treatment.

## 2 Short-chain fatty acids

### 2.1 Introduction to short-chain fatty acids

The main short-chain fatty acids (SCFAs) are acetate, propionate, and butyrate (Feitelson et al., 2023). Acetate is predominantly produced by *Propionibacterium*, while propionate is synthesized by various bacterial taxa, including *Firmicutes*, *Bacteroidetes*, *Acetobacter*, and *Clostridium*. Butyrate is mainly generated by *Firmicutes*. SCFAs play significant roles in modulating disease processes, including carcinogenesis, by attenuating inflammation and regulating host gene expression through diverse mechanisms. SCFAs impede disease progression by inhibiting NF-κB activation, decreasing the production of pro-inflammatory cytokines, increasing anti-inflammatory cytokines, and facilitating the naive T cell differentiation into regulatory T cells (Tregs), which suppress immune responses (Qu et al., 2025). This enhances Treg proliferation and inhibits pro-inflammatory Th17 cells, strengthening protection against colorectal cancer (Karam et al., 2025). Consequently, SCFAs impact CRC development by modulating cytokine profiles and T cell differentiation. Nevertheless, in certain tumor microenvironments, SCFAs may promote tumor growth, depending on tumor type, concentration, and local metabolic and immune conditions. For instance, acetic acid can fuel tumor growth under hypoxia or nutrient scarcity by converting to cholesterol and fatty acids via acetyl-CoA. In contrast, *F. rodentium*-derived acetic acid boosts CD8^+^ T cell immunity and inhibits the PI3K/AKT/mTOR pathway, suppressing tumors. Butyrate exerts dual effects on cell proliferation in a concentration-dependent manner: it stimulates proliferation at low concentrations but suppresses it at high concentrations. The subsequent section describe the molecular mechanisms responsible for this dual, concentration-dependent regulation.

### 2.2 Butyrate is regarded as the most significant SCFAs

Among SCFAs, butyrate stands out as a vital metabolite generated through gut bacterial fermentation, conferring a wide spectrum of health benefits. It exhibits a greater capacity to suppress the proliferation of HT-29 colon cancer cells compared to acetate or propionate. Butyrate inhibits tumors by blocking cancer cell proliferation, inducing cell cycle arrest, and promoting apoptosis (Geng et al., 2021). As a histone deacetylase (HDAC) inhibitor, it triggers cancer cell death and boosts interferon-γ (IFN-γ) production in effector T cells (Arpaia et al., 2013). It also enhances cancer cell death by regulating cytokines and key metabolic pathways (Shao et al., 2021; Wang et al., 2022; Qu et al., 2025). Nonetheless, butyrate exerts both antitumor and pro-tumor properties, which are primarily determined by its concentration.

### 2.3 Inhibitory effect of butyrate on CRC tumorigenesis

As a HDAC inhibitor, butyrate promotes Foxp3 expression and reduces the production of pro-inflammatory cytokines by dendritic cells, thereby favoring the extrathymic differentiation of Tregs. These butyrate-induced Tregs can then reinforce immune tolerance programs, helping to reshape local inflammation and the tumor micro-environment in CRC (Arpaia et al., 2013). *Bacteroides fragilis*, a Gram-negative, obligate anaerobic bacterium, plays a pivotal role in promoting the production of SCFAs, particularly butyrate. Butyrate, in turn, suppresses the NLRP3-mediated inflammatory signaling pathway, thereby attenuating macrophage activation and the release of pro-inflammatory cytokines such as IL-1β and IL-18. This mechanism not only ameliorates intestinal inflammation but also constrains the progression of colitis-associated cancer (CAC) (Xu et al., 2021). By analyzing the gut MEGA database, the authors identified *Bacteroides fragilis* strains with differential abundance. Using a combination of experimental approaches, including murine colorectal tumor model development, broad-spectrum antibiotic depletion of the microbiota, and targeted transplantation of *Bacteroides fragilis*, the study systematically investigates how this bacterium suppresses the NLRP3-mediated inflammatory signaling pathway through enhanced butyrate production, thereby attenuating intestinal inflammation and inhibiting the progression of CAC. On the immune side, butyrate upregulates TRAF5, which binds to and inhibits the dimerization of the gp130 receptor, blocking the activation of the pro-tumor IL-6/JAK2/STAT3 signaling pathway (Ryu et al., 2022). This study directly demonstrates the essential role of TRAF5 in butyrate-mediated signaling through experimental validation at both cellular and *in vivo* levels. By integrating protein stability assays, the work proposes a mechanistic pathway whereby butyrate upregulates TRAF5 expression and enhances its interaction with gp130, thereby promoting gp130 degradation and suppressing oncogenic signal transduction. Future investigations should aim to elucidate the upstream regulatory mechanisms underlying butyrate-induced TRAF5 modulation and validate the clinical relevance of this pathway in patients with CRC.

Butyrate can also suppress tumor growth by modulating microRNA expression profiles and intracellular reactive oxygen species (ROS) levels. For example, it upregulates miR-22, which promotes apoptosis through the inhibition of SIRT1. In turn, SIRT1 suppression results in ROS accumulation, thereby inhibiting the proliferation of CRC cells (Huang et al., 2023). This study established a comprehensive evidence chain through reciprocal validation using gain-of-function and loss-of-function experiments, demonstrating that the miR-22/SIRT1/ROS axis constitutes a key molecular mechanism underlying butyrate-mediated pro-apoptotic effects. However, the core mediating role of miR-22 was confirmed only at the molecular level. Future studies should aim to further validate the direct targeting relationship between miR-22 and SIRT1 and confirm the functional relevance of this signaling pathway in *in vivo* animal models, thereby strengthening its causal basis and potential for clinical translation. Butyrate suppresses CRC through a combination of metabolic and immune mechanisms. On the metabolic front, butyrate markedly inhibits glucose metabolism and DNA synthesis in CRC cells by downregulating the membrane expression of GLUT1 and G6PD via the GPR109a-AKT signaling pathway (Yuan et al., 2020). Geng et al. established a causal role for butyrate in suppressing glucose metabolism through the GPR109a-AKT signaling axis, employing a comprehensive set of gain-of-function and loss-of-function experiments. However, this evidence remains largely confined to molecular mechanistic insights *in vitro*. The physiological dominance of this signaling pathway *in vivo* and its potential for clinical translation require further validation in physiologically relevant animal models and human cohorts.

Butyrate contributes to the reinforcement of the mucosal barrier by enhancing MUC gene expression and promoting mucin production, a key component of intestinal defense. When the mucosal barrier is compromised, as demonstrated in MUC2 knockout mice, colitis can develop and the risk of CRC markedly increases. Additionally, butyrate produced by *Roseburia intestinalis* binds to the TLR5 receptor on CD8^+^ T cells, promoting their activation. Given that TLR5 expression on CD8^+^ T cells is associated with enhanced anti-tumor immunity, this represents a distinct immunomodulatory pathway through which butyrate augments the host’s anti-tumor immune response (Stoeva et al., 2021). This mechanism has been systematically validated across multiple experimental models, confirming that butyrate enhances CD8^+^ T cell function via activation of the TLR5-NF-κB signaling pathway, thereby potentiating anti-tumor immunity in animal models. These findings provide robust mechanistic evidence and causal support for the role of microbial metabolites in regulating anti-tumor immunity. However, interventional clinical trial data from patients with CRC have not yet been included, and thus, the clinical causality in humans remains to be further established.

PLAC8 is a protein critically involved in mitosis and cell cycle regulation. In CRC cells, overexpression of PLAC8 accelerates tumor growth and enhances migratory capacity, thereby potentially increasing metastatic potential. Notably, a recent study on butyrate-producing microbes reported that butyrate selectively downregulates PLAC8 in cells exhibiting aberrantly high expression levels and induces apoptosis, suggesting a potential role for butyrate in modulating CRC cell fate through this pathway (Chang et al., 2022). Basic experimental evidence indicates that the depletion of butyrate-producing bacteria in the gut and the consequent reduction in butyrate levels are associated with CRC progression, with the underlying mechanism involving butyrate-mediated downregulation of the oncogene PLAC8. However, this study did not involve direct supplementation of butyrate or butyrate-producing bacteria in animal models to assess their dynamic effects on PLAC8 expression and tumor growth, nor were interventional studies conducted in human patients. Consequently, the clinical causal relationship between butyrate deficiency and CRC progression remains to be further established.

### 2.4 Promoting effect of butyrate on the development of CRC

Contrary to its anti-tumor properties, accumulating evidence suggests that butyrate may exert tumor-promoting effects under certain conditions. The potential mechanisms underlying this duality may involve variations in butyrate concentration, cellular metabolic states, and immune regulatory pathways. In specific cell populations, butyrate is rapidly metabolized, leading to diminished HDAC inhibitory activity and a shift toward serving as a metabolic substrate that supports cell survival. Such concentration-dependent effects may contribute to this functional switch. Moreover, butyrate’s role in immune modulation does not invariably result in anti-tumor activity; in particular immunological contexts, excessive accumulation of immune cells may drive chronic inflammation, thereby promoting carcinogenesis.

Numerous studies have demonstrated that butyrate can promote CRC progression in various animal models and human observational studies (Irrazabal et al., 2014; Louis et al., 2014). The phenomenon known as the “butyrate paradox”, whereby butyrate exerts both tumor-promoting and tumor-suppressive effects, is commonly attributed to concentration-dependent mechanisms. For instance, low concentrations may stimulate tumor growth, whereas high concentrations tend to inhibit it. Butyrate concentration gradients along the colonic epithelium have been experimentally validated by Donohoe et al. Their findings indicate that the colonic lumen maintains a butyrate concentration of approximately 5 mM. However, due to the thick mucus layer covering the epithelial surface continuously replenished by goblet cells, the concentration reaching the crypt base cells is markedly reduced to approximately 50-800 μM. Under these low-concentration conditions, crypt base cells utilize butyrate as an energy substrate through Acetyl-CoA production and HAT-mediated pathways, promoting beta-oxidation and fueling the tricarboxylic acid (TCA) cycle. This metabolic reprogramming increases Acetyl-CoA availability, upregulates proliferation-associated genes, and ultimately supports cellular proliferation. In contrast, luminal surface cells are exposed to near-maximal butyrate levels (~5 mM) over prolonged periods. Here, butyrate primarily functions as a HDAC inhibitor, leading to the accumulation of acetylated histones, upregulation of apoptosis-related genes, suppression of cell proliferation, and induction of cell death. Notably, cancer cells often display dysregulated metabolism, including the Warburg effect, which impairs their capacity to metabolize butyrate via beta-oxidation. Consequently, even at lower concentrations, butyrate predominantly acts as an HDAC inhibitor in malignant cells, thereby suppressing proliferation and triggering apoptosis. Thus, in low-butyrate microenvironments such as the crypt base, the Acetyl-CoA-HAT axis appears to play a pivotal role in mediating pro-proliferative outcomes in normal colonocytes (C.L.Donohoe et al., 2012). This study elucidated the mechanism underlying the butyrate concentration gradient and revealed how butyrate exerts a “dual function” in the colon depending on its concentration and the metabolic state of target cells. Specifically, at lower concentrations in the crypt base, butyrate serves as an energy substrate for normal colonic epithelial cells by fueling the TCA cycle through beta-oxidation, thereby supporting homeostatic renewal. In contrast, at higher luminal concentrations, it acts predominantly as a HDAC inhibitor in transformed or cancerous cells, leading to histone hyperacetylation, cell cycle arrest, and induction of apoptosis. This functional dichotomy highlights a key metabolic vulnerability in cancer cells with impaired butyrate metabolism, which renders them susceptible to epigenetic regulation even at low concentrations. Consequently, future targeted therapeutic strategies may exploit this differential response to achieve a wider therapeutic window.

SCFAs are widely recognized for their beneficial contributions to colorectal health. Among these, butyrate displays dual and context-dependent functional roles, with its biological effects shaped by local concentration, cellular metabolic capacity, and the immunomodulatory microenvironments. To advance mechanistic understanding, future research must systematically address these multifaceted variables. A clear delineation between pro-tumor and anti-tumor outcomes necessitates rigorous assessment of concentration gradients, tissue-specific distribution, host cell metabolic phenotypes, and immune regulatory networks. Robust preclinical animal models and well-controlled human clinical trials are required to evaluate the therapeutic efficacy of direct butyrate supplementation. Moreover, a comprehensive evaluation of environmental and physiological factors, particularly dosage regimens, delivery methods, and microbial sources of butyrate, is essential to define their influence on *in vivo* bioactivity. Ultimately, a deeper integration of these determinants will be critical for the rational development and clinical translation of butyrate-based strategies in CRC prevention and therapy.

## 3 Bile acid

### 3.1 Bile acids in promoting the development of CRC

Bile acids are synthesized from cholesterol in the liver and are essential for the absorption of dietary fats and fat-soluble vitamins. Beyond their digestive functions, bile acids serve as signaling molecules that regulate metabolism and immune homeostasis. They contribute to the maintenance of gut barrier integrity, modulate the composition of the gut microbiota, and promote intestinal equilibrium via immunomodulatory mechanisms, all of which are implicated in CRC development. The roles of bile acids in CRC are multifaceted and depend on their specific types, concentrations, as well as host microbiota and genetic background. The farnesoid X receptor (FXR), which is highly expressed in intestinal epithelial cells, acts as the primary bile acid receptor. Under physiological conditions, bile acids activate FXR, thereby regulating glucose and lipid metabolism, as well as controlling bile acid synthesis and transport, conferring protective effects (Fuchs et al., 2013; Dawson and Karpen, 2015; Nguyen et al., 2021). In mouse models, FXR deficiency disrupts the gut barrier and increases immune infiltration, promoting CRC. In patients with CRC, APC gene deletion silences the FXR promoter via hypermethylation, downregulating genes like IBABP and upregulating COX-2 and c-Myc, driving tumor progression (Pithva et al., 2015). The transporter ABCC3 (MRP3) is involved in bile acid efflux. Reduced ABCC3 expression results in bile acid accumulation, subsequent activation of MAPK signaling, and enhanced oncogenesis (Turjman and Nair, 1984). Although ABCC3 is shown to affect bile acid levels and MAPK activity, its direct role in CRC development *in vivo* remains unproven. Thus, the therapeutic potential of targeting the ABCC3-bile acid-MAPK pathway needs further *in vivo* validation. Epidemiological evidence associates elevated fecal bile acid (FBA) levels with a heightened risk of CRC. Experimental studies corroborate that bile acids, particularly secondary bile acids, induce cellular damage through mutagenesis, increased cellular turnover, cell lysis, and DNA damage (Li et al., 2022). However, SBAs can also have opposing effects in CRC, contingent upon their specific molecular species.

### 3.2 Dual role of secondary bile acids in the development of CRC

Although Secondary bile acids are commonly present in the gut microbiota, these metabolites exert dual and often opposing effects that are less dependent on concentration than on the specific composition of bile acid species. For instance, deoxycholic acid (DCA) facilitates tumorigenesis through several mechanisms, such as inducing chronic inflammation, stimulating epithelial cell proliferation, and causing direct DNA damage. Similarly, lithocholic acid (LCA) contributes to a pro-tumorigenic environment by upregulating oncogenic signaling pathways such as NF-κB and Wnt/β-catenin. In contrast, ursodeoxycholic acid (UDCA) exhibits protective effects against CRC progression, mediated by anti-inflammatory actions and cytoprotective functions, including inhibition of apoptosis in healthy epithelial cells. Therefore, the net impact of secondary bile acids on CRC pathogenesis is determined by the relative balance among individual molecular species, each conferring distinct and potentially antagonistic biological activities.

**Figure 2:**
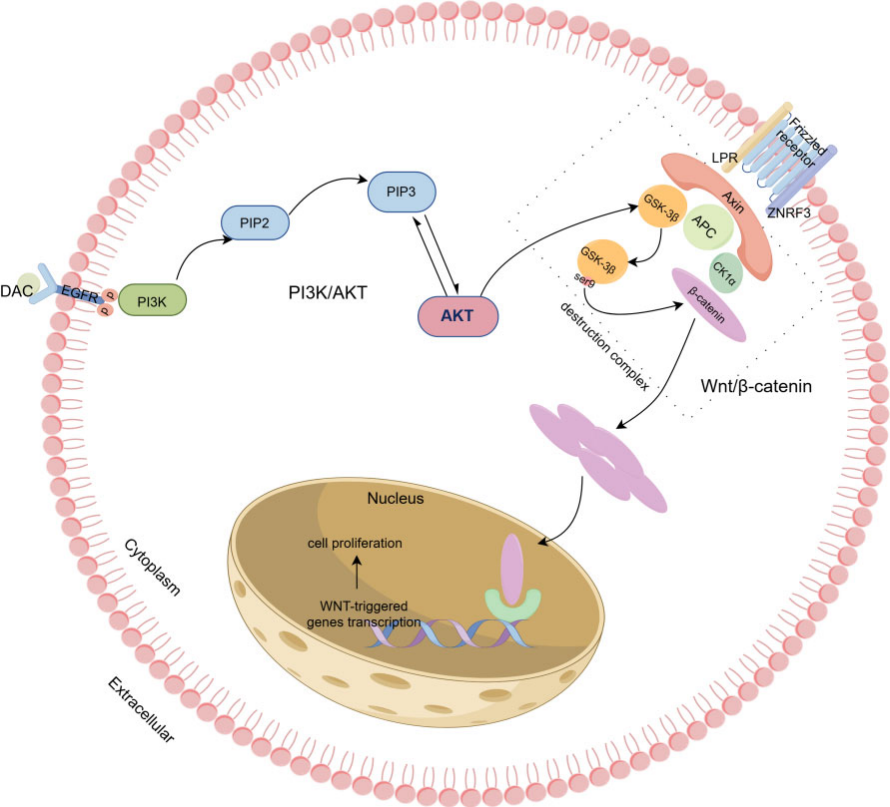
DCA activates the epidermal growth factor receptor (EGFR), initiating downstream signaling cascades such as the PI3K/Akt pathway. Subsequently, PI3K upregulates β-catenin signaling, likely by inactivating glycogen synthase kinase 3β (GSK3B). This inactivation prevents the formation of the β-catenin destruction complex, leading to β-catenin accumulation, activation of Wnt downstream signaling, and ultimately tumorigenesis [35]. From a dietary perspective, a high-fat diet enriches gut microbes like Clostridium, which produce secondary bile acids. These bile acids can disrupt cell mitosis, induce DNA damage, and promote reactive oxygen species (ROS) production. Supporting this, in vitro studies have confirmed that deoxycholic acid promotes the proliferation of human colon adenoma (AA/C1) cells and inhibits their apoptosis, thereby driving colorectal carcinogenesis. By Figdraw.

Secondary bile acids can promote the initiation and progression of CRC. An increase in bile acid-metabolizing bacteria leads to elevated secondary bile acid concentrations, which subsequently upregulate Rspo3 expression in the gut. This upregulation stimulates LGR4, LGR5, and β-catenin expression, thereby activating the Wnt/β-catenin signaling pathway through both LGR-dependent and independent mechanisms (Hilkens et al., 2017), thereby driving CRC development (Jean-Louis et al., 2006). Notably, DCA activates the epidermal growth factor receptor (EGFR), initiating a downstream signaling cascade prominently involving the PI3K/AKT pathway. Activation of PI3K subsequently upregulates β-catenin signaling, likely through inactivation of GSK3β. This inactivation prevents formation of the β-catenin destruction complex, resulting in β-catenin accumulation, activation of Wnt target genes, and ultimately promotion of tumorigenesis (Caliceti et al., 2022) **Figure 2**. *In vitro* studies have demonstrated that DCA promotes proliferation of human colon adenoma (AA/C1) cells while suppressing apoptosis, thus contributing to CRC development and progression (Zhang et al., 2021; Wang et al., 2022). Collectively, these findings provide robust mechanistic evidence that DCA drives CRC through coordinated modulation of key oncogenic pathways. LAC activates the ERK/AP-1 signaling pathway in a dose- and time-dependent manner while simultaneously inhibiting STAT3, thereby stimulating miR-21 promoter activity in HCT116 cells. The upregulation of miR-21 expression precedes the suppression of phosphatase and tensin homolog (PTEN), and enhanced CRC cell proliferation may be mediated through activation of the PI3K/AKT signaling pathway. This study provides substantial mechanistic evidence that LAC promotes CRC cell proliferation via coordinated regulation of key signaling nodes (Sato et al., 2024). In contrast to the tumor-promoting effects of DCA and LCA, UDCA appears to offer protective benefits in CRC. Studies have shown that dietary supplementation with UDCA reduces tumor incidence in the AOM/DSS mouse model of inflammation-driven CRC (Fathima and Jamma, 2024). Mechanistically, Zhang H et al. systematically elucidated the causal mechanism by which UDCA suppresses CRC cell proliferation and tumor growth through multi-level animal experiments. UDCA upregulates TGR5 expression, promotes cAMP accumulation, and activates protein kinase A (PKA), leading to inhibition of RhoA activity, downregulation of YAP expression, and reduced nuclear translocation of YAP. These findings demonstrate a functionally coherent signaling cascade that links TGR5 activation to YAP inactivation, providing mechanistic insights into the anti-tumor effects of UDCA (Candelli et al., 2021).

Although DCA, LCA, UDCA are all products of bile acid metabolism, their roles in CRC are strikingly divergent. DCA and LCA predominantly exert pro-tumorigenic effects by activating oncogenic signaling pathways that drive CRC initiation and progression. In contrast, UDCA demonstrates tumor-suppressive potential, primarily through its ability to modulate anti-inflammatory responses. These opposing actions highlight the delicate balance within bile acid metabolism and underscore how subtle shifts in bile acid composition can shift the equilibrium toward either tumor promotion or suppression. This functional duality not only provides new insights into the role of bile acids in tumor biology but also offers a promising foundation for developing personalized therapeutic strategies. Moving forward, integrated studies examining bile acid concentrations, metabolic pathways, and the local immune microenvironments may pave the way for more precise and effective interventions. Future studies incorporating animal experiments and clinical trials are necessary to establish a comprehensive mechanism-pathology-intervention evidence chain.

## 4 The role of LPS in the pathogenesis and progression of CRC

LPS, a major constituent of the outer membrane of Gram-negative bacteria and commonly referred to as endotoxin, is a potent inducer of inflammatory responses (Yu et al., 2024). Structurally, it comprises three distinct domains: lipid A, core oligosaccharide, and the O antigen (Sulit et al., 2023).

### 4.1 LPS promotes CRC development through the modulation of long non-coding RNAs and histone lysine lactylation

Studies indicate that an increased abundance of Gram-negative bacteria in tumor tissues may drive LPS-mediated alterations in long non-coding RNA (lncRNA) expression. Specifically, LPS elevates lactate levels and induces histone lysine lactylation, thereby modulating gene expression. One notable example is LINC00152, which shows higher expression in clinical CRC samples than in adjacent normal tissues. LPS promotes this overexpression by facilitating histone lactylation at the LINC00152 promoter, reducing the binding of the transcriptional repressor YY1 and consequently enhancing tumor growth (Wu et al., 2021). In a parallel pathway, LPS activates the NF-κB signaling cascade via TLR4 binding, resulting in increased VEGFC production, which further contributes to CRC metastasis (Panyathep et al., 2023).

### 4.2 LPS promote CRC progression through cytokine-mediated inflammation and signaling pathway activation

*Fusobacterium* and *Bacteroides* are the major sources of LPS production in the CRC microenvironments. LPS from *F. periodonticum* and *B. fragilis* upregulates key cytokines, including IL-1β, IFN-γ, IL-18, IL-10, IL-6, and IL-12 p70, which exhibit opposing effects on tumor progression. For example, IL-18 and IL-6 demonstrate potential tumor-suppressive properties, whereas IFN-γ compromises the colonic barrier and facilitates cancer dissemination metastasis (Guangwei et al., 2022). Mechanistically, LPS promotes CRC invasion and migration by triggering the NF-κB/VEGF-C pathway through TRAF6 ubiquitination, thereby stimulating lymphatic vessel formation (Zhu et al., 2016; Firmal et al., 2022). Furthermore, it augments glycolysis and metastasis through the NF-κB/Snail/HK3 axis (Skrzypczak-Wiercioch and Sałat, 2022). Collectively, these findings substantiate a prominent role for LPS in advancing CRC metastasis; however, the evidence remains confined to preclinical models and lacks validation in human studies.

### 4.3 The dual role of LPS in tumor promotion and inhibition in CRC

Although numerous studies have established the tumor-promoting role of LPS, its effects are not exclusively oncogenic. As demonstrated by Firmal P et al., LPS can upregulate TOPORS expression, which in turn modulates STAT1 activity. Concurrently, SMAR1 suppresses STAT3 in T cells, a mechanism linked to favorable clinical outcomes in cancer patients (Shi et al., 2022). This indicates that LPS activity is markedly context-dependent, influenced by specific microenvironmental and cellular conditions. Consequently, additional investigation is necessary to discriminate between the impacts of bacterially surface-anchored LPS and exogenously administered LPS on CRC patient prognosis. Clarifying this distinction is essential for a nuanced understanding of the complex role of LPS in CRC.

## 5 Amino acids and amino acid-related metabolites

Microbial amino acid metabolism, centered on tryptophan, yields secondary metabolites with a substantial influence on CRC progression. The gut microbiota produces a spectrum of tryptophan-derived metabolites capable of activating the aryl hydrocarbon receptor (AHR). Subsequent AHR activation contributes to tumor progression by inhibiting anti-tumor immunity, facilitating immune evasion, inducing angiogenesis, and augmenting therapeutic resistance, thereby collectively promoting tumor growth and metastasis (Sun et al., 2023; Zhang et al., 2023a).

Tryptophan-related metabolites exert multifaceted effects within the tumor microenvironment. The kynurenine pathway represents the primary route through which cells catabolize tryptophan into kynurenine, a metabolite that promotes tumor growth by mediating immune suppression and stimulating angiogenesis. In contrast, the gut microbiota plays a key role by directly metabolizing tryptophan into indole derivatives and related compounds, which may inhibit tumor progression through suppression of immune evasion or induction of apoptosis. Therefore, investigations must integrate a dual-perspective framework that considers both the pro-tumorigenic and anti-tumorigenic functions of these metabolites. A thorough understanding of these countervailing mechanisms is fundamental for a precise assessment of their therapeutic potential in cancer treatment.

### 5.1 The role of tryptophan-related metabolites in inhibiting CRC development

Zhao TL et al. demonstrated through basic research that the inhibitory effect of 5-methoxytryptophan (5-MTP) on CRC cell proliferation via the PI3K/AKT/FoxO3a signaling pathway (Sikder et al., 2020). Walczak K et al., in a preliminary mechanistic study, reported that 8-hydroxyquinolinic acid exhibits anti-proliferative and anti-migratory effects against CRC cells, potentially through modulation of cell cycle regulators such as Cyclin D1 and CDK4, as well as through influencing key components of the Wnt/β-catenin pathway, including β-catenin and E-cadherin. However, both studies remain at the level of initial mechanistic exploration, necessitating further *in vivo* validation and target confirmation to fully elucidate their molecular mechanisms and functional relevance (Adak and Khan, 2019; Walczak et al., 2020). Beyond direct cytotoxicity, tryptophan-related metabolites can also modulate gene expression. For example, Xu and colleagues showed that tryptophan binds to the PIWI domain of Ago2, forming a complex with miR-193a-3p that enhances its activity, thereby suppressing target gene expression and inhibiting CRC liver metastasis (Li et al., 2024; Chen et al., 2025). Li Y et al. provided evidence from both *in vitro* and *in vivo* experiments that indole-3-lactate (ILA), a tryptophan metabolite produced by *Bifidobacterium breve (B. breve)*, activates AHR in macrophages, subsequently triggering the PI3K/AKT signaling pathway and promoting the differentiation of immature inflammatory colonic macrophages into mature, homeostatic phenotypes, thus attenuating CAC (Li et al., 2024). Zhang L et al. further corroborated these findings by demonstrating, through integrated *in vivo* and *in vitro* models, that *Akkermansia muciniphila (A. muciniphila)* suppresses CRC progression by inhibiting the tryptophan metabolism-driven AHR/Wnt/β-catenin signaling axis (Zhang et al., 2023a). Lu Y’s team confirmed that the *Eubacterium limosum* strain El1405CS specifically inhibits CRC cell proliferation *in vitro* by altering cell cycle distribution and inducing apoptosis. In the CT26 syngeneic mouse model, daily administration of live El1405, heat-inactivated El1405, its culture supernatant, or indole-derived compounds significantly suppressed tumor growth. 16S rRNA sequencing revealed that El1405 treatment reshaped the intratumoral microbiome by reducing the abundance of *Enterobacter, Pseudomonas*, and *Staphylococcus*. Moreover, El1405 intervention markedly increased levels of TNF-α, IFN-γ, and CD8^+^ T cells within the tumor microenvironment, while decreasing CD4^+^ T cells, IL-6, IL-10, and TGF-β. Metabolomic analysis confirmed that the anti-tumor effects were mediated by indole metabolites, particularly ILA and indole-3-acetate (IAA). This study systematically demonstrates that *Eubacterium limosum* exerts anti-CRC effects through multiple mechanisms, consisting of the production of anti-tumor metabolites, the modulation of the gut microbial composition, and the remodeling of the tumor immune microenvironment. Nevertheless, the specific signaling pathways activated by these metabolites remain to be fully characterized, and clinical validation in human subjects is still lacking (Miyazaki et al., 2022).

Collectively, these studies highlight that gut microbiota-derived tryptophan metabolites can exert protective effects in CRC by precisely regulating critical signaling pathways.

### 5.2 The role of tryptophan-related metabolites in promoting the occurrence of CRC

The gut microbiota plays a crucial role in regulating the host immune system through tryptophan-related metabolites. Moreover, these compounds can activate the aryl hydrocarbon receptor (AHR), a process that ultimately suppresses anti-tumor immunity. For instance, kynurenine promotes tumor immune evasion as demonstrated in a study by Miyazaki T et al., which showed the activation of the TDO2-kynurenine-AHR signaling pathway facilitates CRC liver metastasis by promoting PD-L1-mediated immune evasion and sustaining cancer stemness (Miyazaki et al., 2022). Similarly, a tryptophan metabolite derived from *Peptostreptococcus anaerobius*, IDA, activates AHR to upregulate ALDH1A3, triggering a metabolic cascade that generates NADH and thereby supports FSP1-mediated reduction of coenzyme Q10, leading to ferroptosis resistance. Genetic inhibition of either AHR or ALDH1A3 disrupts this pathway and suppresses tumor growth, identifying the IDA-AHR-ALDH1A3 axis as a key therapeutic target in ferroptosis-resistant CRC (Cui et al., 2024). These two studies, through multi-level experimental interventions and mechanistic analyses, provide robust evidence supporting a causal relationship; however, further validation in human cohorts is required.

In addition, emerging evidence suggests that certain metabolites within this pathway may promote CRC progression. For instance, tryptophan depletion activates the stress-activated kinase GCN2, impairing T cell-mediated immunity and thereby increasing CRC risk (Walczak et al., 2020). Kynurenine has also been shown to contribute to tumorigenesis by reducing CD8^+^ T cell populations and expanding Tregs, thus fostering an immunosuppressive tumor microenvironment conducive to cancer development (Dmitrieva-Posocco et al., 2022).

Overall, tryptophan-related metabolites play a dual role in CRC, with the potential to either inhibit or promote tumor growth. This duality stems from the complex interactions among tryptophan-related metabolites, the immune system, and the tumor microenvironment. For instance, certain metabolites such as 5-MTP and 8-hydroxyquinolinic acid suppress tumor growth through direct cytotoxic effects. In contrast, indole derivatives inhibit CRC progression by blocking key signaling pathways, including PI3K/AKT and AHR/Wnt-β-catenin. However, other metabolites, such as kynurenine and tryptophan depletion, promote tumor progression by facilitating immune evasion and immunosuppression. Therefore, the dual nature of tryptophan-related metabolites indicates that therapeutic strategies targeting the tryptophan metabolic pathway must be precisely tailored. It is essential to consider the specific biological actions of these metabolites and the contextual influence of the tumor microenvironment to minimize potential adverse effects and maximize their anti-tumor efficacy.

### 5.3 The dual roles of other amino acid metabolites in CRC development

In addition to tryptophan metabolites, numerous other amino acids and their metabolites play important roles in in host physiology.

L-arginine, an essential amino acid, plays a key role in tumor development by modulating immune responses. In mouse models of CRC treated with fasting-mimicking diets (FMD), elevated levels of L-arginine, a functional metabolite produced by *Bifidobacterium pseudolongum*, were observed. Further investigations revealed that L-arginine promotes the induction of a tissue-resident memory T cell (TRM) phenotype both *in vivo* and *in vitro*. Mechanistically, L-arginine is transported into CD8^+^ T cells via the SLC7A1 transporter. In CRC patients undergoing FMD treatment, increased abundance of *Bifidobacterium pseudolongum* and higher L-arginine levels were associated with expansion of the CD8^+^ TRM cell population. Notably, elevated levels of CD8^+^ TRM cells and *Bifidobacterium pseudolongum* correlated with improved prognosis in CRC patients (Nan et al., 2025). This study establishes a multi-level evidence chain spanning from animal models to clinical observations, from gut microbiota to immune cells, and from metabolic products to receptor signaling pathways, thereby proposing a novel strategy for combined microbiota-immune therapy in CRC.

In a metabolomics analysis of stool samples from CRC patients, Zhang et al. identified agmatine, a neuroprotective arginine metabolite that is minimally produced endogenously and closely associated with the gut microbiota. In mouse models, the *Shigella flexneri C.11* strain was shown to upregulate pro-inflammatory cytokines (IL-6, IL-1β) and serum tumor markers (CEA, alpha-fetoprotein), indicating its role in promoting intestinal inflammation and accelerating carcinogenesis. This strain enhances CRC tumorigenesis through agmatine production, which induces DNA damage in intestinal epithelial cells. The underlying mechanism involves increased ERBB3-Nrg1 binding, leading to activation of the PI3K/AKT/mTOR signaling pathway and subsequent malignant transformation of normal intestinal epithelial cells, evidenced by altered cell morphology, enhanced proliferation and migration, and dysregulated cell cycle progression (Zhang et al., 2025). In a complementary study, Lu Yu et al. analyzed enriched metabolites in CRC model mice and found that microbially derived agmatine, produced by *Blautia, Odoribacter, Alistipes*, and *Paraprevotella*, interacts with Rnf128 to suppress Rnf128-mediated ubiquitination of β-catenin. This stabilization of β-catenin leads to upregulation of its downstream targets, including Cyclin D1, Lgr5, CD44, and c-Myc, thereby activating Wnt signaling. Activated Wnt signaling promotes intestinal epithelial dysplasia and lymphocytic inflammatory infiltration by inducing upregulation of pro-inflammatory cytokines (IL-6 and TNF-α) and downregulation of the anti-inflammatory cytokine IL-10, thus contributing to CRC (Lu et al., 2024). Together, these two studies provide strong evidence that agmatine is a key functional molecule linking gut microbiota dysbiosis to CRC development. Although this association has not yet met the highest standard of human epidemiological causality, its potential as an early-warning biomarker and therapeutic target is highly significant, offering a clear direction for future clinical translational research.

Targeting key enzymes in the tryptophan pathway has emerged as a promising strategy to inhibit tumor growth. Clinical evidence from CRC patients shows reduced serum tryptophan levels and altered tryptophan metabolites and enzymes-including elevated IL4I1, KYNA, KMO, IDO1, TDO2, 5-HT, and TPH1 in tumors-strongly correlated with clinical parameters, highlighting the critical role of tryptophan metabolism in CRC (Fong et al., 2023; Yu et al., 2024a). As research uncovers the complex links between metabolism and tumorigenesis, investigating the dual roles of metabolites in CRC has become a key focus. Their context-dependent effects remain unclear, so future studies must clarify the underlying molecular mechanisms. Understanding how metabolites influence cancer progression through diverse signaling pathways will enable more sophisticated therapies that target not only cancer cells but also metabolic networks that promote or suppress tumor growth.

## 6 The mechanism and immune interaction of emerging metabolites in CRC

Beyond the well-characterized SCFAs, secondary bile acids, and amino acid-related metabolites, human intestinal tissues harbors a broad spectrum of other metabolites that significantly influence CRC pathogenesis. The following section summarizes the roles of these additional metabolites in CRC development.

### 6.1 Other gut metabolites in promoting CRC onset

In CRC, upregulation of FADS1 enhances arachidonic acid (AA) production and promotes the enrichment of Gram-negative gut microbiota. This microbial shift activates the TLR4/MYD88-NF-κB signaling pathway in CRC cells, initiating a downstream cascade that upregulates PTGS2 and PTGES expression, thereby increasing prostaglandin E2 (PGE2) synthesis. Through this mechanism, the FADS1-AA axis establishes a pro-tumorigenic microenvironment that drives PGE2 accumulation and tumor progression (Xu et al., 2023). These findings reveal a multilevel and intricate causal network. Future therapeutic strategies should focus on synergistic interventions targeting FADS1 enzyme activity, modulation of the gut microbiota, and inhibition of the TLR4/PGE2 signaling axis. Another microbial metabolite 4-hydroxyphenylacetic acid (4-HPA) drives tumor progression by activating the JAK2/STAT3 pathway, resulting in increased CXCL3 expression and subsequent recruitment of polymorphonuclear myeloid-derived suppressor cells (PMN-MDSCs), which suppress anti-tumor CD8^+^ T cell activity (Liao et al., 2025). These results offer strong causal evidence from preclinical models; however, they do not establish definitive epidemiological causality in human populations. Sphingosine kinase 1 (SPHK1) promotes CRC invasion and metastasis by regulating TRAF6-dependent autophagy via ULK1 ubiquitination. The SPHK1/TRAF6 axis is upregulated in CRC and correlates with metastatic progression, highlighting its potential as a therapeutic target. Through functional assays, mechanistic dissection, and rescue experiments, this study not only identified key molecular events but also established a coherent causal framework, providing solid experimental support for the SPHK1-TRAF6-ULK1 axis in CRC pathogenesis (Chen et al., 2024). Additionally, succinate derived from *Fusobacterium nucleatum* impairs the cGAS-STING-IFN-β signaling pathway, reducing Th1-associated chemokine expression within tumors. This leads to diminished CD8^+^ T cell infiltration and weakened anti-tumor immunity, thereby contributing to resistance to PD-1 antibody immunotherapy. Collectively, these findings delineate a clear mechanistic link between gut microbial metabolites and immune evasion in CRC, though clinical confirmation through prospective trials is still required. Trimethylamine-N-oxide (TMAO) promotes intestinal tumorigenesis by activating Wnt/β-catenin signaling through inhibition of the FXR-FGF15 axis. This is demonstrated by TMAO-induced proliferation and VEGFA secretion in CRC cells, as well as enhanced tumor growth and angiogenesis in mouse models with diet-induced elevation of TMAO levels (Yang et al., 2022; Chen et al., 2024). Deng et al. demonstrated that 5-aminovaleric acid (5-AVA), produced by *Fusobacterium mortiferum*, promotes CRC growth by binding to KDM6B, impairing the demethylation of DKK2, and consequently activating the Wnt/β-catenin signaling pathway (Deng et al., 2025). Martin Gallausiaux C et al. provided robust mechanistic support for multiple signaling pathways through multi-level genetic and pharmacological interventions in cellular models. *Fusobacterium nucleatum* releases ADP-heptose into the tumor microenvironment, activating the ALPK1/TIFA/TRAF6 signaling axis in intestinal epithelial cells. This cascade induces NF-κB activation, leading to upregulation of the inflammatory cytokine IL-8 and the anti-apoptotic genes BIRC3 and TNFAIP3-both of which are implicated in CRC. While these findings are compelling, further validation in animal models and clinical settings remains necessary (Martin-Gallausiaux et al., 2024). The robust research design and methodology provide high-quality associative evidence and offer in-depth insights into the underlying molecular mechanisms. These findings not only guide future research directions but also identify promising candidate targets for potential therapeutic interventions.

### 6.2 Other gut metabolites in inhibiting CRC development

β-hydroxybutyrate (BHB) effectively suppresses CRC growth through multiple molecular pathways. BHB activates the cell surface receptor HCAR2, leading to upregulation of the transcriptional regulator HOPX and subsequent modulation of gene expression that inhibits cell proliferation (Martin-Gallausiaux et al., 2024). In a CAC model, exogenous BHB supplementation has been shown to reduce tumor burden and angiogenesis (Dmitrieva-Posocco et al., 2022). Transcriptomic analyses reveal that BHB downregulates VEGFA expression in CAC tumor mucosa. Mechanistically, BHB directly targets the transcription factor HIF-1α, thereby suppressing VEGFA expression in hypoxic CT26 cells. Collectively, these findings demonstrate that BHB inhibits angiogenesis in CAC by modulating the HIF-1α/VEGFA signaling axis (Huang et al., 2024). Based on current evidence, BHB could be regarded as an effective inhibitor of CRC, including CAC. In a study by Yang et al., supplementation with *Faecalibaculum rodentium (F. rodentium)* suppressed CRC in AOM/DSS-induced mouse models and human CRC cell lines (SW620 and HT-29). GC-MS analysis identified acetate as the key tumor-suppressive metabolite, and exogenous acetate administration significantly reduced tumor volume and weight in CRC models. Mechanistically, *F. rodentium*-derived acetate downregulates podoplanin (PDPN) on CD8^+^ T cells and CLEC-2 on tumor cells, thereby enhancing CD8^+^ T cell immunity and inhibiting the PI3K/AKT/mTOR signaling pathway (Yu et al., 2024a). Through a complete experimental framework of “depletion-supplementation-rescue”, this study precisely identified and validated acetate as the sole functional mediator through which *F. rodentium* exerts its anti-tumor effects, establishing a clear causal relationship. Microbial analysis by Bell HN et al. revealed that *Lactobacillus reuteri* and its metabolite reuterin are downregulated in both murine and human CRC. Reuterin exerts anti-tumor effects by disrupting cellular redox homeostasis, thereby reducing the proliferation and survival of CRC cells. Mechanistically, reuterin induces selective protein oxidation, which inhibits ribosome biogenesis and global protein translation, ultimately suppressing tumor growth (Bell et al., 2022). Through multi-model experiments, interventional validation, and an integrated multi-omics evidence chain, this study strongly confirms that *Lactobacillus reuteri* in a healthy gut microbiota and its metabolite reuterin directly inhibit CRC growth by inducing oxidative stress and impairing protein translation. Tyrosol, a gut microbiota-derived metabolite, effectively inhibits tumor development *in vitro* through multiple mechanisms (Peng et al., 2021). The depletion of the beneficial intestinal bacterium *Faecalibacterium prausnitzii* leads to reduced levels of its associated metabolite tyrosol. Tyrosol suppresses CRC growth by inhibiting the NF-κB/HIF-1α signaling pathway, alleviating oxidative stress and inflammatory responses, and promoting CD8^+^ T cell infiltration. *Lactobacillus plantarum* supplementation promotes the production of conjugated linoleic acid (CLA), which exerts multifaceted anti-tumor effects. Strain CCFM8661 activates the PPAR-γ receptor through CLA generation, thereby inhibiting the NF-κB inflammatory pathway, upregulating MUC2, Claudin-1, and ZO-1, repairing the intestinal barrier, promoting tumor cell apoptosis, and alleviating CRC. Fecal microbiota transplantation and metagenomic analyses further reveal that elevated CLA levels enrich the butyrate-producing bacterium *Odoribacter splanchnicus*. This microbial remodeling increases butyrate production, strengthens tight junction proteins, modulates cytokine expression, and improves gut microbiota composition, collectively contributing to CRC prevention (Li et al., 2024; Fan et al., 2025). Although this study presents a comprehensive mechanistic cascade with clinical correlations, the key causal relationships remain primarily supported by inhibitor-based experiments and lack genetic validation, such as gene knockout. In a study by Jaiswal et al., 4-ethylphenylsulfate (4-EPS) exhibited selective anticancer activity against colorectal adenocarcinoma HCT-116 cells, while sparing normal CCD-841 colon epithelial cells (Martin-Gallausiaux et al., 2024). In HCT-116 cells, 4-EPS significantly reduced cell proliferation, viability, ATP levels, and colony formation, while promoting apoptosis. Mechanistically, it upregulated Bax, downregulated Bcl-2, and induced G2/M phase arrest. These findings demonstrate the selectivity of 4-EPS and highlight its potential as a promising therapeutic agent for CRC (Yang et al., 2025). However, the current evidence is limited to phenotypic associations and preliminary mechanistic hypotheses, lacking direct causal validation, which necessitates further investigation through mechanistic studies and animal models **Table 1**. As shown in **Table 1**, the mechanisms of action of some gut microbiota and their metabolites in colorectal cancer are summarized (Gao et al., 2024; Li et al., 2025; Jiang et al., 2025; Tran et al., 2020; Zhang et al., 2023; Zhao et al., 2023).

**Table 1:** Mechanism of Action Of Certain Gut Microbiota and Their Metabolites in Colorectal Cancer.

Microorganisms	Oxygen Demand	Gram Stain	Metabolite	Participation Mechanisms	Role on CRC	Reference
Roseburia intestinalis	parthenogenetic anaerobic	Gram-positive	short-chain fatty acid	Increased pro-apoptotic proteins Caspase-3 and Bax and decreased anti-apoptotic protein Bcl-2	inhibitory	Shao et al., 2021
Bacteroides fragilis	specialized anaerobic	Gram-negative	short-chain fatty acid	Inhibition of macrophage activation and secretion of pro-inflammatory mediators such as IL-18 and IL-1β	inhibitory	Wang et al., 2022
Mucinophilic Ackermannia	anaerobic	Gram-negative	short-chain fatty acid	Metabolizing mucins into acetic and propionic acids, short-chain fatty acids can work with microbes and intestinal cells to promote epithelial barrier function	inhibitory	Huang et al., 2023
Eubacterium	specialized anaerobic	Gram-positive	short-chain fatty acid	Reduced serum IL-6 levels while suppressing tumor size and volume	inhibitory	Oncel et al., 2024
Roseburia intestinalis	anaerobic	Gram-positive	butyrate	Binds TLR5 receptors on CD8+ T cells and promotes CD8+ T cell activation	inhibitory	Stoeva et al., 2021
butyrate-producing bacteria	anaerobic	Gram-negative	butyrate	Down-regulated expression of placental-specific protein 8 (PLAC8) and induced apoptosis in PLAC8 overexpressing cells	inhibitory	Chang et al., 2022
Porphyrinomonas spp.	anaerobic	Gram-negative	butyrate	Induction of cellular senescence	facilitation	Nan et al., 2025
symbiotic clostridium	anaerobic	Gram-positive	branched-chain amino acids	Promotes cholesterol synthesis and activates the Sonic hedgehog signaling pathway	facilitation	Zhang et al., 2023a
Bifidobacterium	anaerobic	Gram-positive	Indole-3-lactic acid	attenuating the pro-inflammatory response of macrophages by activating the PI3K/AKT signaling pathway.	inhibitory	Zhao et al., 2023
Escherichia coli	parthenogenetic anaerobic	Gram-negative	Escherichia coli	L-tryptophan prevents copper toxicity and triggers gene expression without affecting clb gene transcription and inhibits ClbP (colicin-activated peptidase)	inhibitory	Gao et al., 2024
Streptococcus anaerobeus	anaerobic	Gram-positive	trans-3-Indoleacrylic acid	Upregulation of ALDH 1A 3 expression	facilitation	Li et al., 2025
Enterococcus faecalis	parthenogenetic anaerobic	Gram-positive	guanidine	Interacts with Rnf128 and inhibits the degradation of β-catenin, leading to downregulation of IL-10 and upregulation of IL-6 and TNF-α.	facilitation	Zhang et al., 2023b
Fusobacterium nucleatum	anaerobic	Gram-negative	Metabolites with ADP-heptose properties	Increased expression of BIRC3 and TNFAIP3-related anti-apoptotic genes	facilitation	Zhang et al., 2023b
Enterococcus faecalis	parthenogenetic anaerobic	Gram-positive	Biliverdin (BV)	Activates PI3K/AKT/mTOR signaling pathway and increases VEGFA expression and IL-8 secretion	facilitation	Cui et al., 2024
Fusobacterium nucleatum	anaerobic	Gram-negative	succinic acid	Impairment of cGAS-mediated IFN-β signaling pathway, reduction of Th 1-type chemokine expression in tumors, and inhibition of CD8+ T cell migration into the tumor microenvironment	inhibitory	Tran et al., 2020
Lactobacillus reuteri	parthenogenetic anaerobic	Gram-positive	Reuterin	Alteration of redox balance to reduce proliferation and survival of colon cancer cells	inhibitory	Tran et al., 2020
Lactobacillus plantarum	microaerobic	Gram-positive	Conjugated linoleic acid	Inhibition of NF-κB signaling pathway and pro-inflammatory cytokines, upregulation of ZO-1, Claudin-1 and MUC 2	inhibitory	Fan et al., 2025

## 7 Gut microbiota and metabolites in CRC: from mechanisms to therapeutic applications

The dynamic interplay between the gut microbiota, their metabolites, and the host has emerged as a pivotal determinant in CRC, influencing disease progression to treatment development. Dysbiosis of the gut microbiome and alterations in its metabolic output can reshape the tumor microenvironment and modulate immune responses, positioning these components as promising targets for novel diagnostic tools and therapeutic strategies.

Targeting amino acid metabolites constitutes a compelling therapeutic approach for CRC. Inhibiting USP14, a post-translational regulator of IDO1, downregulate IDO1, disrupts tryptophan metabolism, and restores T cell function by reducing Treg generation and boosting CD8^+^ T cell activity. This enhances antitumor immunity and synergizes with anti-PD-1 therapy, offering an alternative to direct IDO1 inhibition (Aganja et al., 2024). In APC-deficient CRC, TDO2 drives tumor growth via the kynurenine pathway. By depleting tryptophan and accumulating kynurenine, TDO2 hosts an immunosuppressive environment that promotes tumor-associated macrophage differentiation and impairs T cell function. The TDO2-kynurenine-AHR axis supports tumorigenesis through both cell-intrinsic and extrinsic mechanisms, rendering it a viable target in precision oncology (Yu et al., 2024b). Dysregulated amino acid metabolism in CRC reveals key therapeutic opportunities. The Wnt pathway upregulates SLC6A14, and its inhibition by α-methyltryptophan (α-MT) induces amino acid starvation, suppresses mTOR, activates autophagy and apoptosis, and inhibits tumor proliferation and invasion, validating SLC6A14 as a drug target (Fathima and Jamma, 2024). In APC-mutant cells, glutamine restriction hyperactivates Wnt signaling due to reduced intracellular α-ketoglutarate (αKG), promoting adenocarcinoma development. Conversely, αKG supplementation normalizes Wnt signaling, improves differentiation, and suppresses tumor growth, identifying αKG as a potent antitumor metabolite (Fathima and Jamma, 2024).

Therapeutic strategies targeting oncogenic signaling pathways show promise in CRC. Kobelt D et al. identified MACC1 as a key downstream target of MEK1, with MEK1-mediated tyrosine phosphorylation essential for MACC1-driven cell motility and metastasis. MEK1 inhibitors block this phosphorylation and reduce MACC1-induced proliferation, indicating that targeting the MEK1-MACC1 axis may suppress tumor progression and metastasis and improve survival (Wong et al., 2022). In APC/KRAS-mutant CRC, enhanced cholesterol biosynthesis is driven by PCSK9 and related genes. Metabolomic analysis shows upregulation leads to geranylgeranyl pyrophosphate (GGPP) accumulation in KRAS-mutant cells and Apc^min/+^ Kras^G12D/+^ Villin-Cre mouse tumors. The PCSK9-GGPP axis directly activates the KRAS/MEK/ERK pathway, highlighting PCSK9 as a therapeutic target in this CRC subtype (Dong et al., 2024). Chondroitin-6-sulfate (C-6-S) activates JAK/STAT3 and Hedgehog signaling, promoting M2 macrophage polarization and creating an immune-rejective microenvironment in microsatellite stable (MSS) CRC. This C-6-S-mediated immunosuppression enables immune escape, suggesting C-6-S as a potential immunotherapeutic target for MSS CRC (Huang et al., 2024).

Therapeutic strategies for CRC based on gut microbiota modulation show significant promise. Haiting Xu et al. developed an oral nanomedicine, LR-S-CD/CpG@LNP, using live *Limosilactobacillus reuteri (LR)* for its cargo delivery and intrinsic immunostimulatory properties. A ROS-responsive nanoplatform is conjugated to the bacterial surface. After oral administration, LR-S-CD/CpG@LNP generates cytotoxic ROS *in situ*, triggering immunogenic cell death in CRC cells. Released tumor neoantigens and CpG oligonucleotides synergistically promote dendritic cell maturation. These mature dendritic cells, aided by LR-secreted metabolites, drive infiltration of tumor-specific cytotoxic T lymphocytes, leading to effective CRC eradication. This approach offers a novel and practical strategy for oral CRC treatment (Kobelt et al., 2021; Papadimitriou et al., 2021). Attenuated *Salmonella*, known for its tumor-targeting and anticancer activity, is a promising microbial therapy candidate. Aganja RP et al. engineered plasmid pJHL90, in which a quorum-sensing promoter controls therapeutic toxin expression. Using tryptophan-auxotrophic, facultative anaerobic *Salmonella strains* with pagL and rfaL deletions, they created a safe, tumor-selective system. The resulting strain expressing cytolysin A (ClyA) enhanced tumor cell killing by 67% versus controls, demonstrating that attenuated *Salmonella* can serve as an effective and biosafe vector for targeted delivery of cytolytic proteins in cancer therapy (Yue et al., 2020). Cell-free metabolites from the probiotic yeast *Saccharomyces boulardii* (SBM) induce apoptosis in human CRC cells and suppress key pro-survival and inflammatory mediators, including survivin, IL-8, and NF-κB, highlighting SBM’s potential as a therapeutic or preventive agent for CRC (Zhang et al., 2024). Metabolites from *Lactobacillus rhamnosus GG*, *Lactobacillus casei M3*, and *Lactobacillus plantarum YYC-3* collectively inhibit the VEGF-MMP pathway, suppressing tumor proliferation and reducing CRC metastasis (Wu et al., 2022). Notably, *Bifidobacterium breve* CCFM683-a CLA-producing strain significantly prevents CRC in mouse models. Its protection is mediated by CLA-induced PPAR-γ upregulation, which inhibits cancer cell proliferation and migration while promoting differentiation and apoptosis. Intestinal CLA also modulates cytokine profiles, suppresses NF-κB signaling, enhances tight junction proteins and MUC2, and improves gut microbiota composition. Together, these findings support *Bifidobacteria* as strong candidates for clinical development in CRC prevention (Gao et al., 2023).

Other therapeutic approaches for CRC target key molecular and microbial pathways. In a study by Zou S et al., CSN6-a subunit overexpressed in various cancers was found to stabilize DDX5 by inhibiting β-Trcp-mediated polyubiquitination and degradation. This stabilization enhances nucleotide metabolism and PHGDH mRNA stability, promoting tumorigenesis. Notably, the microbial metabolite butyrate acts as a functional antagonist of CSN6, highlighting its therapeutic potential in modulating this pathway (Huang et al., 2024). Sodium butyrate functions as both an HDAC and BMI-1 inhibitor, showing strong promise for treating CRC liver metastasis (CRCLM). BMI-1 drives CRCLM by promoting epithelial-mesenchymal transition (EMT) and metastasis via activation of the AKT/GSK-3β/Snail pathway. Thus, targeting the BMI-1 oncogenic axis interaction, exemplified by sodium butyrate, offers a promising strategy for CRCLM therapy (Papadimitriou et al., 2021; Bayne et al., 2024). Recent studies identify Pien Tze Huang (PZH), a traditional Chinese medicine containing musk, cow bezoar (Niuhuang), snake gall, and Panax notoginseng (Sanqi), as a potent modulator of the CRC gut microenvironment. PZH treatment reduces dysbiosis by enriching beneficial bacteria such as *Pseudobutyrivibrio xylanivorans* and *Eubacterium limosum* and increasing advantageous metabolites like taurine, hypotaurine, bile acids, and unsaturated fatty acids. This microbial restoration improves intestinal barrier function and suppresses oncogenic signaling (Zhang et al., 2023a). Butyrate from *Roseburia intestinalis* binds TLR5 on CD8^+^ T cells, activating NF-κB signaling to enhance T cell activity and antitumor immunity. This mechanism boosts anti-PD-1 efficacy in mice with MSI-low CT26 tumors, positioning *R. intestinalis* as a promising probiotic to overcome immune checkpoint blockade resistance in cancer patients (Kang et al., 2023).

## 8 Clinical translation challenges and potential solutions for microbiota-targeted metabolite therapy

This article has highlighted the pivotal role of the gut microbiota and their metabolites in the pathogenesis and progression of CRC, a perspective strongly supported by physiological mechanisms and multi-omics evidence from extensive literature. However, translating these insights into metabolite-targeted therapies faces significant challenges, primarily due to profound inter-individual variability. The efficacy of a given metabolite often depends on specific bacterial consortia for its production. Yet, even among individuals with similar microbial compositions, functional gene repertoires and metabolic capacities can vary markedly as a result of diet, environmental exposures, and host genetics. Moreover, functional redundancy in the gut ecosystem-where multiple distinct bacterial species can produce the same metabolite-complicates interventions targeting individual taxa.

Beyond microbial heterogeneity, the bioavailability of metabolites represents another critical determinant of therapeutic success. Efficacy observed *in vitro* frequently fails to translate to clinical outcomes, as bioavailability is influenced by diverse factors, including race, individual physiology, digestive enzymes, and gastrointestinal transit time. A prominent example is oral butyrate, which is predominantly absorbed or metabolized in the upper gastrointestinal tract and thus rarely reaches the colorectum at therapeutically effective concentrations. Therefore, future research must prioritize strategies to overcome these barriers, such as designing precursors or stabilized derivatives of key metabolites, or leveraging advanced biotechnologies like targeted encapsulation systems to protect compounds from premature degradation and ensure site-specific delivery.

The safety implications of targeting microbial metabolites also warrant careful consideration. Exogenous administration of a metabolite may disrupt the homeostasis of the gut ecosystem, potentially promoting the overgrowth of pathogens or opportunistic microbes. Furthermore, systemic effects on non-target organs pose significant concerns. For instance, elevated plasma levels of TMAO are associated with increased risks of inflammation, endothelial dysfunction, type 2 diabetes, and cardiovascular diseases. TMAO exemplifies how a single metabolite can exert detrimental distal effects, underscoring the necessity for comprehensive safety assessments in the development of metabolite-based therapies (Gou et al., 2023).

To facilitate the clinical translation of the utilization of gut microbial modulation, we propose several practical strategies. Tryptophan metabolites exhibit contrasting roles in CRC. Microbial indole derivatives maintain intestinal barrier integrity and inhibit cancer cell proliferation, exerting anti-tumor effects. In contrast, kynurenine, mainly produced by host cells, accumulates during chronic inflammation or in the tumor microenvironment, suppressing immune surveillance and promoting tumor growth. To leverage this duality for prevention, it is recommended to improve patients’ diet and use probiotics to boost protective indole production; engage in regular physical activity and maintain healthy body weight to reduce inflammation, On a therapeutic and mechanistic level, targeting specific signaling pathways and gut microbiota serves as promising candidates, such as inhibit IDO activity, limiting kynurenine pathway activation, and apply fecal microbiota transplantation from donors enriched with indole-producing bacteria to reshape the gut microbiota and increase beneficial metabolite levels. Butyrate also shows dose-dependent duality. At low levels, it activates GPR43/41 and may promote tumorigenesis; at high levels, it inhibits HDACs, induces apoptosis, and exerts anti-tumor effects by modulating the tumor microenvironment. To precisely control such metabolites, integrating multi-omics technologies via metabolomics can be used to monitor butyrate levels and combine metagenomics with transcriptomics to build predictive models. This approach enables targeted interventions for CRC prevention and supports monitoring during radiotherapy and chemotherapy. Similarly, fecal microbiota transplantation can be used to shift secondary bile acid metabolism by introducing microbes that convert primary bile acids into UDCA. This increases protective UDCA and reduces pro-carcinogenic bile acids.

Oral administration of beneficial commensals can suppress CRC development and improve treatment outcomes. Beyond probiotics, traditional Chinese medicine formulations also promote beneficial bacteria and metabolites. Nanotechnology enables precise microbiome modulation. By using bacterial-targeting ligands, drug carriers can be directed to tumors, allowing *in situ* modulation of the gut flora (Wei et al., 2024). For example, the engineered nanomedicine LR-S-CD/CpG@LNP induces immunogenic cell death, activates dendritic cells, and promotes cytotoxic T lymphocyte infiltration after oral administration, leading to tumor eradication in CRC (Yue et al., 2020). Integrating 16S rRNA sequencing, targeted metabolomics, and proteomics helps identify key microbes and metabolic markers linked to CRC. Machine learning models built on large-scale multi-omics data can reveal critical regulatory factors in disease development. This approach supports precise subtyping and individualized therapies, improving both efficacy and safety in clinical practice.

## 9 Discussion

This article highlights the dual roles of key microbial metabolites in CRC pathogenesis. A balanced gut microbiota and its metabolic products are essential for intestinal homeostasis. In contrast, microbial dysbiosis and metabolic imbalances, especially overproduction of harmful metabolites, can disrupt intestinal function, leading to immune dysregulation, impaired epithelial barrier, increased microbial translocation, and chronic inflammation. These processes collectively promote CRC development and progression (Salvi and Cowles, 2021).

SCFAs, bile acids, and tryptophan metabolites, though distinct in structure and origin, collectively exert a dual influence within the tumor microenvironment. This duality is driven by a combination of context-dependent signaling, cell-type specificity, and the intrinsic properties of each metabolite. For instance, butyrate can enhance anti-tumor immunity through HDAC inhibition and Treg differentiation, yet may be co-opted by tumor cells as an energy source via Warburg-like metabolic pathways. Secondary bile acids foster colorectal carcinogenesis by activating the Wnt/β-catenin pathway in intestinal epithelial cells, whereas UDCA counters tumor proliferation by triggering TGR5 and PKA signaling in tumor cells. Similarly, select tryptophan metabolites support anti-tumor immune responses by activating PI3K/AKT signaling in macrophages, while IAA can activate the aryl AHR and ALDH1A3, conferring resistance to ferroptosis and promoting tumor progression. The tryptophan metabolic pathway not only mediates immunosuppression and angiogenesis, facilitating cancer development, but also serves as a central hub in the AHR signaling axis, integrating tryptophan metabolism, immune regulation, and tumor immune evasion. Ultimately, the biological impact of these metabolites is determined by their local concentrations, relative balance, the composition of the microbiota, and the host cellular context, underscoring the complex interplay between microbial metabolism and cancer progression.

Given that the dual effects of SCFAs, bile acids, and tryptophan metabolites are intricately shaped by local concentrations, microbial composition, and host cellular context-reflecting the complex crosstalk between microbial metabolism and cancer progression-deciphering these context-dependent interactions and translating them into targeted microbial interventions requires the integration of multi-omics technologies, which can unravel the interplay between gut microbiota and host gene expression at the molecular level. Integrating host transcriptomics and microbiome data, known as interactomics, helps elucidate these interactions at the gene level. Qin et al. combined 16S rRNA and metagenomic sequencing of mucosal communities, linking *Enterobacteriaceae* to changes in bile acid secretion and CD4^+^ effector memory T cells (CD4^+^ Tem), and found *Mycobacterium tuberculosis* enriched in tumor mucosa, suggesting a role in CRC development (Qin et al., 2025). Dai Z. et al., in a CRC metagenome meta-analysis, showed that GO/KO pathways linked to *Bacteroides fragilis* were largely independent of other CRC-related bacteria, indicating a unique role for *B. fragilis* in CRC (Qin et al., 2025). Multi-omics methods are key to understanding the gut microbiome’s role in CRC. Zou S. et al. integrated fecal microbiome data with tumor and normal tissue genomics and transcriptomics, showing *Fusobacterium nucleatum* may act via the TNFSF9 pathway to alter the tumor immune environment and drive cancer (Adak and Khan, 2019). Zhang S.L. et al. used the DIABLO algorithm on multi-omics data and found *Lactobacillus* correlated with higher CXCL9 level, which recruits effector T cells and boosts anti-tumor immunity (Zhang et al., 2023). Together, these results show multi-omics integration reveals how tumor-associated microbial function. Future studies should combine metabolomics and proteomics to better understand CRC mechanisms. These advances improve knowledge of microbial metabolites in CRC and support developing new metabolite-based treatments, offering strong potential to enhance CRC diagnosis, prognosis, and therapy.

By unraveling the functional crosstalk between the gut microbiota and host genes in CRC via multi-omics integration, these mechanistic insights not only advance our understanding of microbial regulation in tumor progression but also play a critical foundation for translating microbial metabolite research into clinical applications-where these metabolites hold substantial promise for augmenting conventional cancer therapies and overcoming treatment limitations in CRC. Butyrate supplementation sensitizes CRC cells to 5-fluorouracil (5-FU) (Li et al., 2024) and helps overcome resistance to immune checkpoint inhibitors (Zhang et al., 2023a). A clinical study from Qingdao University found that butyrate reduces colorectal adenoma recurrence, supporting its preventive role. Combining microbial metabolites with standard treatments offers a promising strategy to improve CRC outcomes. The gut microbiota and its metabolites are increasingly recognized for their potential to enhance anti-tumor immune responses and improve the efficacy of chemotherapy, radiotherapy, and immunotherapy in CRC patients-an emerging frontier in microbiome-based interventions. Translating microbial metabolites from preclinical findings to clinical applications requires rigorous validation, optimization, and personalized adaptation. A synergistic integration of basic research and well-designed clinical trials is essential to ensure both safety and therapeutic efficacy. Initially, the safety profile of microbial metabolites should be established through Phase I clinical trials, followed by Phase II studies to evaluate efficacy, and ultimately confirmed in large-scale, randomized Phase III trials. Throughout this translational pipeline, close monitoring of treatment-related adverse effects, dynamic changes in patient biomarkers, and comprehensive efficacy assessments is critical. Given that microbial metabolites may elicit variable responses across individuals, personalized treatment strategies are indispensable during clinical development. By integrating genomics, metabolomics, and microbiome profiling, clinicians can tailor optimal therapeutic regimens for individual patients, thereby improving treatment outcomes and minimizing adverse effects. Furthermore, continuous collection of clinical data and long-term patient follow-up are imperative to evaluate sustained efficacy, long-term safety, and the potential emergence of unforeseen side effects over time.

While the clinical translation of microbial metabolites demands rigorous validation and personalized adaptation, the inherent dual role of these metabolites in CRC further enables a paradigm of precision modulation for cancer therapy-one that leverages targeted microbiota manipulation, metabolite regulation and multi-omics-driven stratification to optimize treatment efficacy and safety. Modulating gut microbiota, adding tumor-suppressive metabolites, or blocking tumor-promoting pathways can improve therapy. Multi-omics data help build models to track dose-dependent metabolites like butyrate, maintaining optimal levels may enhance chemotherapy through multiple mechanisms. Phage therapy can selectively remove bacteria that produce cancer-promoting metabolites, reshaping the gut microbiota and improving chemotherapy tolerance. Personalized interventions based on fecal metabolomic and genomic profiles can increase treatment precision and safety. For example, inhibiting *Peptostreptococcus anaerobius* while adding beneficial bacteria that produce indole derivatives allows bidirectional control of tryptophan metabolism. Combining multi-omics data with clinical factors, such as age, sex, disease stage, subtype, markers, and comorbidities, helps identify patient subgroups with different treatment responses. Analyzing tumor and normal tissues reveals the intratumoral microbiome structure, while microbial DNA from stool, oral swabs, or blood can aid non-invasive cancer detection. Microbiome-based biomarkers are key for early screening, predicting treatment response, and prognosis, supporting personalized therapy (Guo et al., 2024). Probiotics like *Lactobacillus rhamnosus* (SCFA producer) and *Bifidobacterium breve* (CLA producer) can boost anti-tumor immunity and improve immunotherapy outcomes. To elucidate the dual role of gut microbiota metabolites in the immune microenvironment of CRC, a multi-layered research approach is essential, spanning from macro-level correlations to micro-level mechanisms and from associative observations to causal validation. Integrating genomics, transcriptomics, proteomics, and metabolomics enables the identification of molecular subtypes and key driver pathways in CRC, facilitating the construction of an integrated network that links microbial metabolites with immune cell profiles and identifies potential therapeutic candidates. Next, co-culture systems combined with molecular biology techniques can be employed to investigate how these metabolites directly modulate immune cell functions and to dissect the underlying signaling pathways. Finally, *in vivo* studies using microbiome-and metabolite-modulated mouse models allow for the validation of causal relationships and assessment of therapeutic potential. Furthermore, single-cell multi-omics technologies, such as the integration of single-cell RNA sequencing with ATAC-seq or proteomic profiling, provide unprecedented resolution into tumor heterogeneity and the complex cellular interactions within the tumor microenvironment. This comprehensive strategy will significantly advance our understanding of the intricate interplay between gut-derived metabolites, the immune system, and CRC progression.

Although clinical evidence is still limited, integrated therapies have strong potential to improve patient outcomes. Future research should focus on using multi-omics to understand how specific probiotic strains, microbial metabolites, and the host immune system interact, guiding the rational design of microbiome-based cancer treatments. Key priorities include identifying effective combination regimens and ensuring safety to support clinical use.

## 10 Limitation

While this review outlines the role of microbial metabolites in CRC, several methodological limitations in the current field must be acknowledged. While such research provides key insights into molecular and cellular processes, it often fails to reflect the full complexity of human physiology and disease. First, most mechanistic insights come from preclinical mouse models, which do not fully reflect the genetic, microbial, and tumor microenvironmental complexity of human CRC (Gates et al., 2025). Cell models also cannot replicate the complex microenvironment or inter-tissue communication seen *in vivo*. Despite genetic similarities, human immune regulation and cognitive functions are more advanced. Human diseases develop over decades, whereas animal models show rapid progression. Patient-specific factors like genetic variation, comorbidities, and environmental exposures are difficult to fully reproduce experimentally. Second, the tumor immune microenvironment and direct interactions between gut microbes and immune cells in CRC progression and treatment response were not a central focus. To address this, multi-omics approaches integrating metabolomics, proteomics, and genomics should be used. Experimental tools such as fecal microbiota transplantation, germ-free animal models, and *in vitro* co-culture systems can help clarify how microbes regulate immune function. Multimodal bioinformatics can further dissect the molecular mechanisms of microbe-immune interactions. Third, although strong associations exist between microbial metabolites and CRC development, a more comprehensive understanding requires combining metabolomics with immune profiling to study tumor metabolic reprogramming at a systems level (Chen et al., 2025).Finally, safety is critical when translating microbial metabolites into clinical use. Mechanistic studies often use surrogate endpoints, such as reduced tumor size that may not correlate with clinical benefits like longer survival or improved quality of life. Therefore, caution is needed when applying preclinical findings to clinical practice. This involves rigorous toxicological testing, dose-escalation studies *in vitro*, in animals, and in early-phase trials. Attention must also be paid to metabolite sources, contamination risks, and active safety monitoring, including for allergic reactions and drug stability *in vivo*. Manufacturing processes must meet regulatory standards. However, current agencies lack a dedicated framework for evaluating microbial metabolite-based therapies, creating major barriers to clinical translation. Addressing these gaps is essential for developing safe and effective treatments.
