# Supplementary material for: Gut microbial metabolites in colorectal cancer: dual roles in tumorigenesis, immune crosstalk, and therapeutic innovation
Source: Front Cell Infect Microbiol. 2026 May 28;16:1693161. doi: 10.3389/fcimb.2026.1693161 (PMC13253271; doi:10.3389/fcimb.2026.1693161)

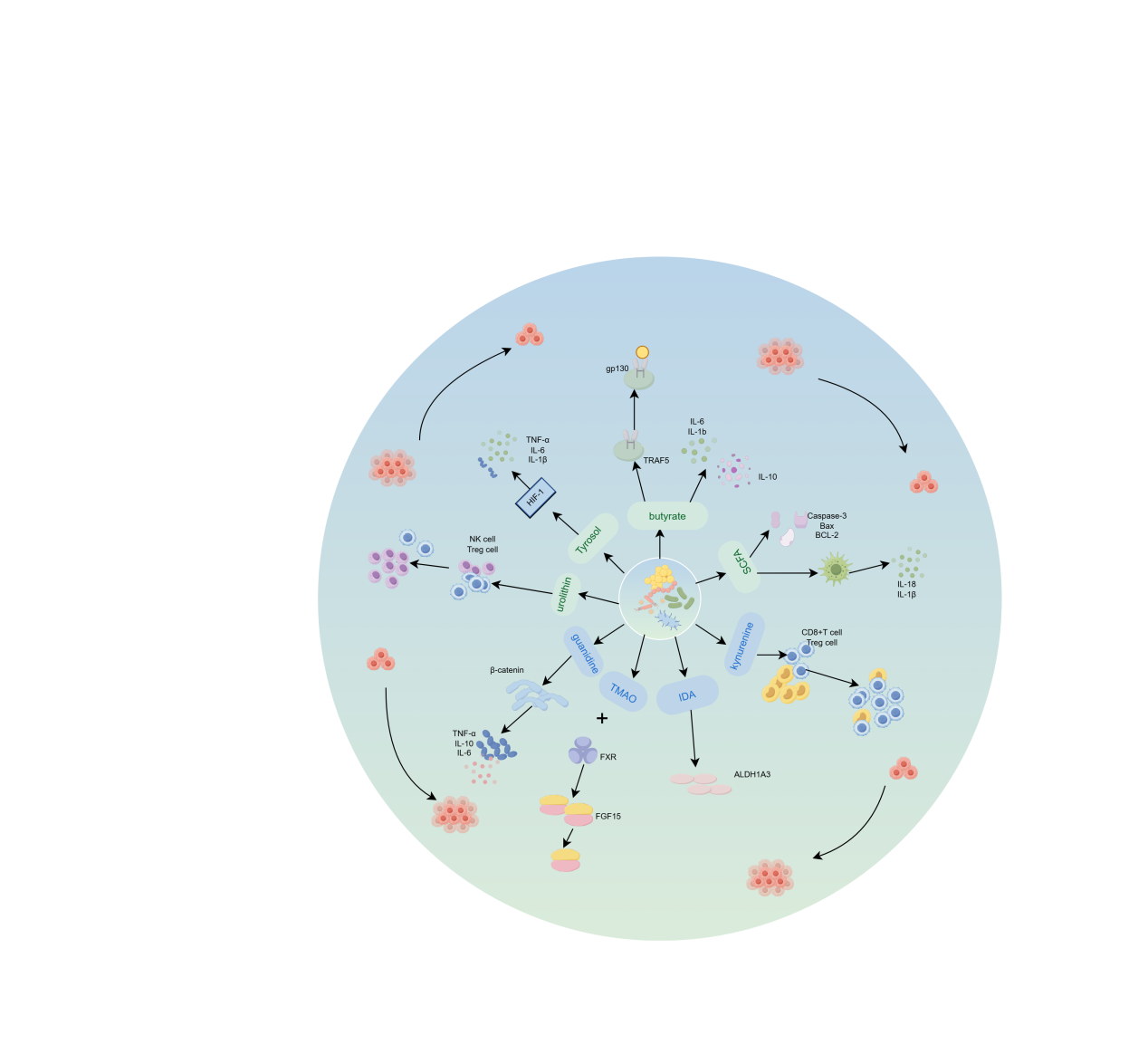


Figure 2 The metabolites of gut microbiota are closely associated with the development of colorectal cancer. The figure below illustrates the relationship between some metabolic pathways and colorectal cancer.（1）Metabolic Pathways Inhibiting the Onset of Colorectal Cancer：1)Lactobacillus reuteri produces short-chain fatty acids (SCFAs), increases the expression of pro-apoptotic proteins Caspase-3 and Bax, decreases the expression of anti-apoptotic protein Bcl-2, and thereby inhibits the onset of colorectal cancer；2）Butyrate can inhibit the expression of pro-inflammatory factors IL-1β and IL-6, while promoting the expression of anti-inflammatory cytokine IL-10, thereby exerting anti-inflammatory effects and increasing the apoptosis of cancer cells；3）Butyrate can upregulate the level of TRAF5 and enhance the binding of TRAF5 to gp130, thereby inhibiting the dimerization of gp130. This inhibition further suppresses the activation of the IL-6/JAK2/STAT3 signaling pathway and ultimately increases the apoptosis of cancer cells。4）Tyrosol can activate the HIF-1 signaling pathway, and at the same time, it can reduce the expression of cytokines such as TNF-α, IL-6, and IL-1β, thereby inhibiting tumor progression；5）Urolithins can increase the numbers of NK cells and γδ T cells in the colorectal tumor microenvironment, while inhibiting the number of Treg cells, and ultimately exert an anti-tumor effect。（2）Metabolic Pathways Promoting Colorectal Cancer Development：1）TMAO can bind to the farnesoid X receptor (FXR), inhibit the FXR-fibroblast growth factor 15 (FGF15) axis, activate the Wnt/β-catenin signaling pathway, and thereby enhance the carcinogenic effect on the intestine.；2）Biliverdin can activate the PI3K/AKT/mTOR signaling pathway, increase the expression of vascular endothelial growth factor A (VEGFA), and promote the secretion of interleukin-8 (IL-8), thereby facilitating tumor growth；3）IDA promotes the development of colorectal cancer by activating the aryl hydrocarbon receptor (AHR), which in turn increases the binding affinity of AHR to the region approximately 100 base pairs (bp) upstream of the transcription start site of aldehyde dehydrogenase 1 family member A3 (ALDH1A3).；4）Guanidine can inhibit the degradation of β-catenin, which leads to the downregulation of interleukin-10 (IL-10) and the upregulation of interleukin-6 (IL-6) and tumor necrosis factor-α (TNF-α). This cascade of events results in the excessive proliferation of intestinal cells and thereby promotes the progression of colorectal cancer (CRC)。By Figdraw。


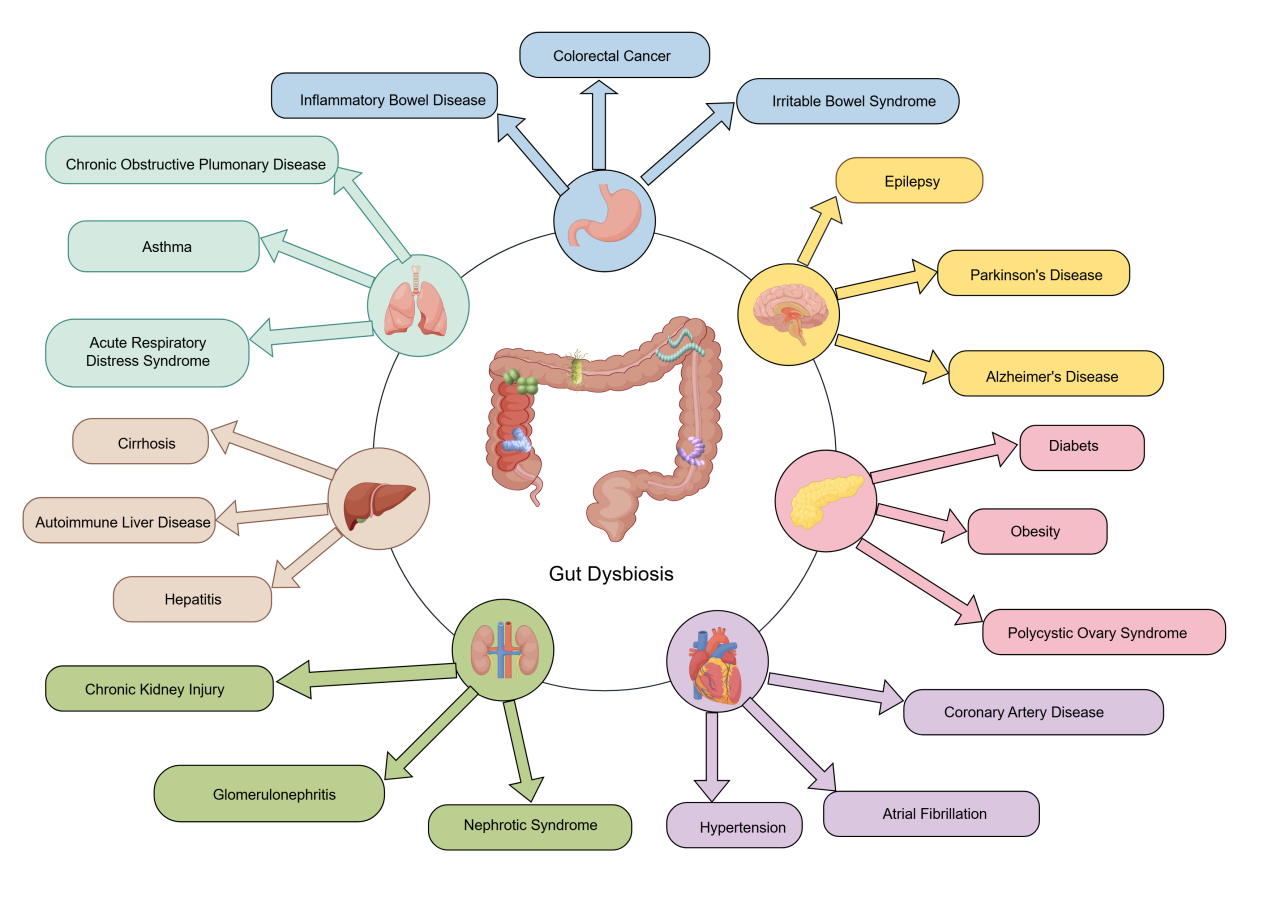


Figure 3 Human health is closely linked to the gut microbiota.Alterations in the intestinal microbiota are called dysbiosis. Dysbiosis interferes with the functioning of the microbiota and can have varying sizes of effects on various organs in the body, leading to a variety of diseases such as colorectal cancer, Parkinson's disease, glomerulonephritis, coronary artery atherosclerotic heart disease, and autoimmune liver disease.By Figdraw。


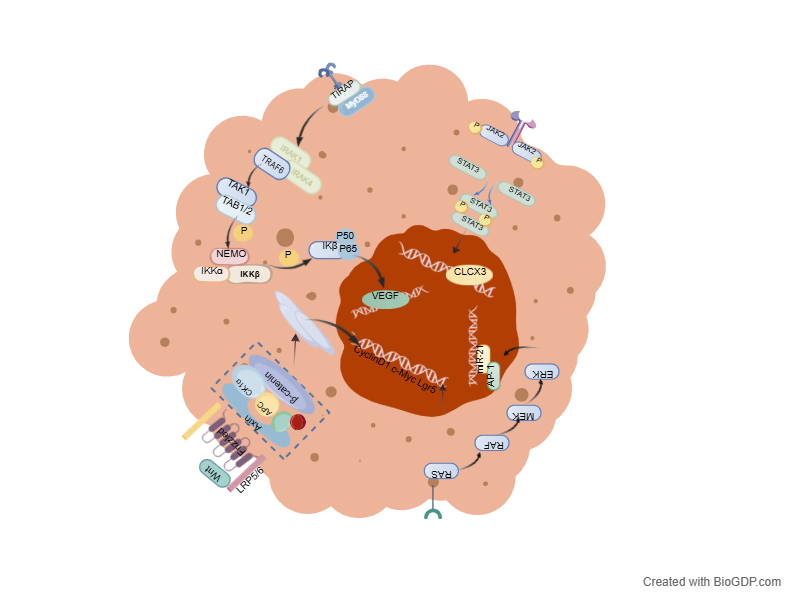


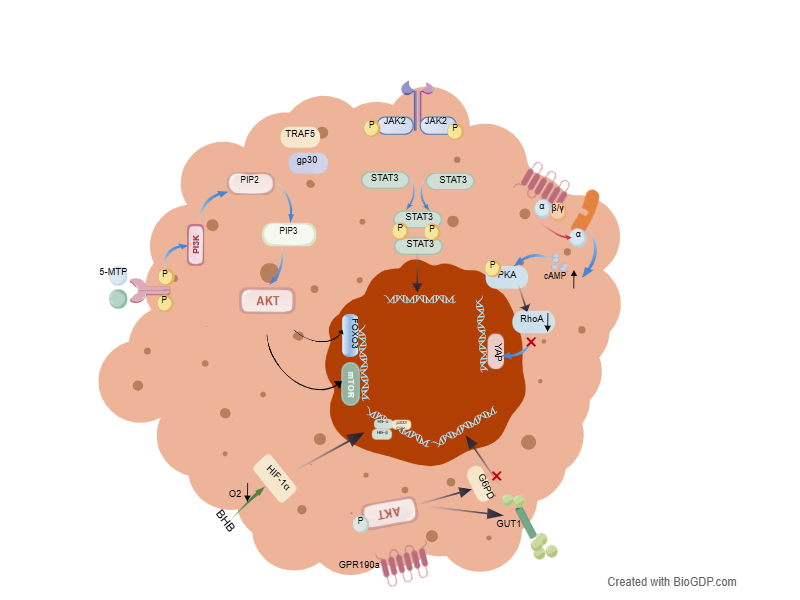


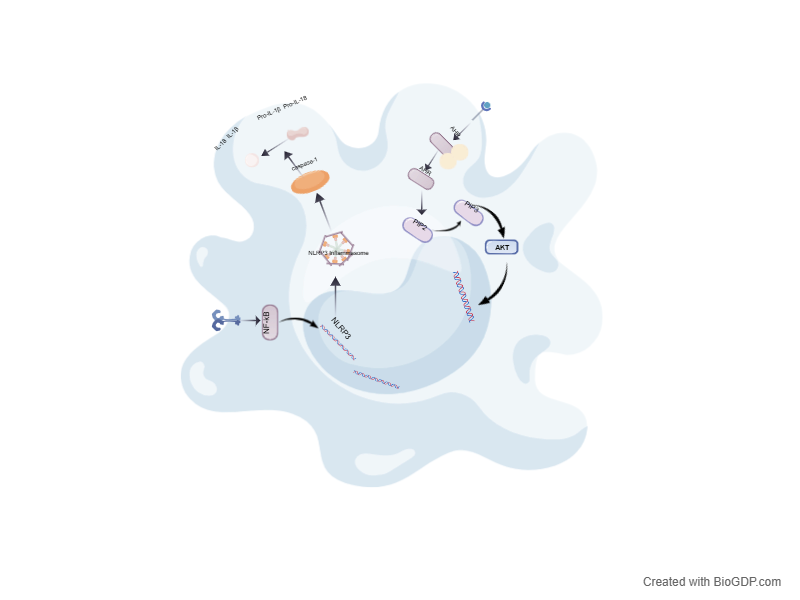


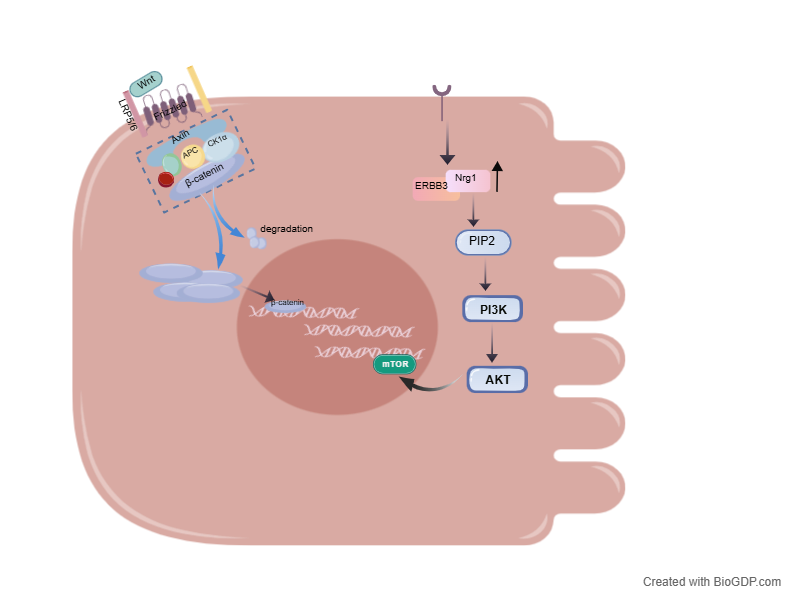

Supplement: Supplementary file 1 [file SupplementaryFile1.docx]
